# Physical Activity Patterns of People Affected by Depressive and Anxiety Disorders as Measured by Accelerometers: A Cross-Sectional Study

**DOI:** 10.1371/journal.pone.0115894

**Published:** 2015-01-13

**Authors:** Björg Helgadóttir, Yvonne Forsell, Örjan Ekblom

**Affiliations:** 1 Department of Public Health Sciences, Karolinska Institutet, Stockholm, Sweden; 2 The Åstrand Laboratory of Work Physiology, the Swedish School of Sport and Health Sciences, Stockholm, Sweden; University of Iowa Hospitals & Clinics, UNITED STATES

## Abstract

**Background:**

Exercise can relieve both depressive and anxiety disorders and it is therefore of importance to establish movement patterns of mildly to moderately affected sufferers to estimate the treatment potential. The aim is to describe the physical activity patterns of people affected by mild to moderate depressive and/or anxiety symptoms using objective measures of physical activity.

**Methods:**

The design of the study was cross-sectional using data from 165 people aged 18–65 years, with mild to moderate depressive and/or anxiety disorder symptoms (scoring ≥10 on the PHQ-9). Diagnoses were made using Mini International Neuropsychiatric Interview (MINI) and symptom severity was measured with the Montgomery-Åsberg Depression Rating Scale (MADRS). The participants wore accelerometers for a week to evaluate physical activity patterns.

**Results:**

No statistically significant differences were detected between different diagnoses, though depressed participants tended to be less active and more sedentary. Only one-fifth of the sample followed public health guidelines regarding physical activity. Each one point increase in MADRS was associated with a 2.4 minute reduction in light physical activity, independent of moderate-to-vigorous physical activity and sedentary time. MADRS was positively associated with number of sedentary bouts.

**Conclusions:**

The physical activity pattern of people with depressive and/or anxiety disorders was characterized by large amounts of sedentary time and low fulfillment of physical activity guidelines. There is therefore a large treatment potential for this group by increasing exercise. The results suggest that instead of focusing exclusively on high intensity exercise for treating depressive and anxiety disorders, health care providers might encourage patients to reduce sedentary time by increasing light physical activity and decreasing the number of sedentary bouts, though further studies are needed that can determine directionality.

## Introduction

Both depressive and anxiety disorders can affect psychomotor functioning, such as gross and fine motor activity, speech characteristics, and motor speed[1,2]. There is a growing amount of evidence to support exercise as a way of reducing the symptoms of depressive and anxiety disorders[3,4] especially for those affected by mild to moderate depression. Conversely, knowledge about physical activity patterns, and therefore the potential for treatment in this group remains limited. An association between depression and increased sedentary behavior as well as decreased exercise has previously been established with questionnaire data[5,6]. However in the last few decades, accelerometers have emerged as a practical option to objectively measure physical activity. The advantages include avoidance of social desirability bias and being able to better estimate sedentary and light activity, and measuring the nuances of physical activity such as bouts of activity and breaks in sedentary time[7–9]. A review of previous research using activity monitors has shown that depressed patients have less motor activity in the daytime than their healthy counterparts[10]. These studies were mainly performed on hospitalized patients and examined how activity levels differed throughout the day. One study has used accelerometers to describe the physical activity patterns of depressed individuals during one week. This study found that people with more depressive symptoms had lower levels of light and moderate physical activity, compared to people with less or no depressive symptoms[11]. Even less research has been done on the physical activity patterns of people suffering from anxiety disorders, though some of the symptoms, such as restlessness, irritability, muscle tension and difficulties concentrating[12] could lead to higher levels of physical activity than that of the general population and even those who suffer from depressive disorders.

Previous accelerometer studies have shown that the benefits of physical activity to somatic health are not solely due to moderate or vigorous physical activity but that even lighter forms of activity can have benefits for health when compared to being sedentary[13]. Not only has it been established that large amounts of sedentary time can be detrimental to a person’s physical health[14,15] but it also matters how the sedentary time is accrued, as extensive sedentary bouts, i.e. few breaks in sedentary time, can be linked to risk of metabolic disease[16,17].

Few accelerometer based studies have been conducted in depressed populations and to our knowledge no previous studies have explored multiple aspects of physical activity using measures such as sedentary bouts and activity bouts to assess the physical activity patterns of a non-clinical population composed of people suffering from depressive or anxiety disorders using well-defined diagnosis-based groups. Since physical activity has been shown to relieve the symptoms of depression and anxiety, it is of importance to establish movement patterns of mildly affected sufferers to estimate the treatment potential. Differences between these groups and the general population may also be of importance when designing preventive interventions and for the provision of care to patients.

The aims of the present study were to 1) describe the physical activity patterns of people affected by mild to moderate depressive and/or anxiety disorders to determine treatment potential and 2) explore whether symptom severity is associated with activity pattern. The aims were achieved by using accelerometer data and we believe that the results will be of interests of clinicians who want to advise their patients as well as be of use to public health officials in the construction of guidelines regarding changes in physical activity patterns for the treatment of depressive and anxiety disorders.

## Materials and Methods

The data for this study is derived from the Regassa project (www.regassa.se), a randomized controlled trial conducted in six Swedish counties and regions. Participants aged 18–65 and having a score of ≥10 on the Patient Health Questionnaire (PHQ-9)[18] were recruited through advertisements and at primary health care centers from 1^st^ of February 2011 to 31^st^ of January 2013. PHQ-9 is reported to be a valid way of diagnosing depressive disorders, a score of ≥10 has been shown to have a sensitivity of 88% and specificity of 88% for major depression[19]. Exclusion criteria included alcohol or drug dependency and abuse, serious somatic disorders, or psychiatric disorders in need of specialist treatment. The participants were randomized into three groups: exercise, internet-based cognitive behavioral therapy and treatment as usual. The present study only included data from the exercise arm of the study (n = 308), as they were the only group to wear accelerometers.

The Mini International Neuropsychiatric Interview (MINI) was used to diagnose psychiatric disorders. The MINI is both reliable and valid when comparing diagnoses of mental disorders with the DSM-III-R and ICD-10[20]. The results were used to categorize participants into having a depressive disorder (major depressive episode, dysthymia), anxiety disorder (panic disorder, social phobia, post-traumatic stress disorder, generalized anxiety disorder) or comorbid disorder (having at least one depressive and one anxiety disorder). The Montgomery-Åsberg Depression Rating Scale (MADRS) was used to assess the severity of depressive symptoms[21].

Triaxial accelerometers (ActiGraph GT3X+) were used to measure the physical activity of the participants. The advantage of using triaxial accelerometers as opposed to uniaxial versions is that they capture not only “up and down” movement but also “back-and-forth” and “side-to-side” activity. Participants were instructed to wear an accelerometer on the right hip, continuously throughout the waking part of the day, removing it only for bathing or swimming and to wear them for seven days before their exercise treatment began. Actilife version 6.4.3 was used for the processing and analyzing the accelerometer data. Monitors were initialized to give average data on movements in 60 second epochs. All the data was checked manually for spuriously high values (caused by electrical interference or malfunction of the accelerometer) and those days that showed evidence of this were removed (n = 1). The accelerometer data was used only if the participants wore the accelerometer for four or more days, including at least one weekend day, which is a widely used method of representing a week of physical activity[22]. Only days with ≥600 minutes of wear time per day were included in the analysis[22]. Non-wear periods were defined as ≥60 minutes of no activity (0 counts/minute), allowing for short bouts (≤2 minutes) of activity above this threshold. Of the 308 participants, 165 fulfilled these requirements (53.6%).

Each person’s total amount of physical activity was summed into average counts per minute. The activity counts were also divided into sedentary activity (<100 counts/min)[23], light physical activity (LIPA, 100–1951 counts/min) and moderate to very vigorous physical activity (MVPA, ≥1952 counts/min)[24]. Sedentary bouts were defined as being sedentary (<100 counts/min) for a minimum of 20 minutes and were presented as number of sedentary bouts and total time in sedentary bouts. Activity bouts are defined as ≥10 minutes in MVPA with an allowance of 2 minutes of interrupted time where the counts could drop below 1952 counts/min. This is a good way of estimating whether a person has met physical activity guidelines as it is recommended that physical activity be performed in bouts of at least 10 minutes[25]. Each of these measures was calculated as the average for each person for the days that they wore the accelerometer. In addition to these seven continuous variables, two categorical accelerometer variables were constructed. Activity bouts were categorized into those fulfilling the public health guidelines of ≥30 minutes of MVPA in ≥10 minute bouts each day of the week or those that did not. We also compared those that fulfilled these requirements ≥5 days a week and those that did not.

Several variables were considered as possible confounders. These included sex and age (continuous), and body mass index (BMI, continuous, based on self-reported height and weight). In addition health status was derived from questionnaire data and was categorized into having 0, 1, or ≥2 serious health conditions that were being treated by a physician at the time of inclusion in the study. Eleven health conditions were listed and the most common were reduced mobility, rheumatologic disorders, back, shoulder or neck pain (14.0%), headache (10.7%), and cardiovascular disease, including high blood pressure (8.4%). Other conditions were less common (neurological disease, diabetes or other metabolic disease, asthma or other lung conditions, ulcer and other chronic conditions of the digestive system and the liver, diseases in kidneys, urine tract or uterus, serious infection or injury, tumor or other serious disease). In addition to this, physical activity in the year preceding the inclusion in the study was derived from self-report data using a single question to categorize people into inactive, doing some light physical activity and regular exercisers. This variable was only used to check for differences in those that fulfilled our accelerometer requirements (wearers) and those that did not (non-wearers).

### Statistical analysis

Differences in characteristics between wearers and non-wearers of accelerometers were explored using Chi^2^ tests and t-tests. There were no statistical differences in sex (Chi^2^ = 1.38, p = 0.239), MADRS scores (t = −0.14, p = 0.891), diagnosis (Chi^2^ = 4.18, p = 0.124), health status (Chi^2^ = 5.80, p = 0.055), or physical activity levels (Chi^2^ = 4.35, p = 0.114) between those that fulfilled the accelerometer requirements and those that did not (see Table 1). However, those that did not have sufficient wear time were on average younger (t = −2.61, p = 0.009) and had lower BMI (t = −2.80, p = 0.006) compared to those that had sufficient wear time.

**Table 1:** Characteristics of the samples, divided into wearers and non-wearers of accelerometers.

	**All**	**Wearers**	**Non-wearers**
	**Mean (SD)**	**Mean (SD)**	**Mean (SD)**
**Age**	41.83 (11.62)	**43.42 (11.42)**	**39.98 (11.62)**
**MADRS**	22.15 (6.98)	22.20 (6.89)	22.09 (7.10)
**BMI**	25.47 (4.13)	**26.07 (3.88)**	**24.76 (4.31)**
**Sex**	% (n)	% (n)	% (n)
***Male***	29.87 (92)	32.73 (54)	26.57 (38)
***Female***	70.13 (216)	67.27 (111)	73.43 (105)
**Diagnosis**			
***Depressive disorders***	12.33 (37)	12.88 (21)	11.68 (16)
***Concurrent disorders***	77.33 (232)	73.62 (120)	81.75 (112)
***Anxiety disorders***	10.33 (31)	13.50 (22)	6.57 (9)
**Health status**			
***0 somatic disorders***	68.18 (210)	63.64 (105)	73.43 (105)
***1 somatic disorder***	19.48 (60)	20.00 (33)	18.88 (27)
***≥2 somatic disorders***	12.34 (38)	16.36 (27)	7.69 (11)
**Physical activity**			
***Inactive***	16.61 (51)	18.18 (30)	14.79 (21)
***Light physical activity***	45.93 (141)	49.70 (82)	41.55 (59)
***Moderate to vigorous exercise***	37.46 (115)	32.12 (53)	43.66 (62)

Numbers in **bold** represent statistically significant results (p<0.01) from two-tailed t-tests or Chi^2^ analyses.

Each continuous accelerometer variable was checked for normality by comparing the mean to the median, and using the Shapiro-Wilk test for normality. Average counts per minute, total time in sedentary bouts, activity bouts and MVPA were found to be statistically different from normal with the Shapiro-Wilk test for normality and activity bouts. Each of these variables was then logged (using the natural logarithm) and all analyses were done both with the logged and original versions of the variables. As the results were not materially different, the findings from the analysis with original versions are displayed.

The relationship between each of the seven continuous accelerometer variables and diagnosis (MINI) was tested with one-way ANOVAs and the differences between categories were explored using the Bonferroni correction for multiple comparisons. As Levene’s test of equality of variance was significant for number of sedentary bouts across diagnoses p = 0.045), the Welch correction was used to adjust for unequal variances, though F-tests yielded similar results for the unadjusted and the adjusted. ANCOVA was used to adjust for sex, age, BMI and health status but associations remained virtually unchanged, thus the unadjusted results from the ANOVAs that are displayed. T-tests were used to explore the differences in the means of the continuous accelerometer variables between the sexes. Adjustment for age, symptom severity (MADRS), BMI, health status was made using ANCOVA but only the unadjusted associations are shown as adjustment had no material effect on the results. Chi^2^ tests were used to analyze the relationships between the two categorical activity bouts variables on the one hand, and sex and diagnosis on the other. The association of each of the continuous accelerometer variables to MADRS was explored using multiple linear regression models, controlling for sex, age, BMI, health status and MVPA. SAS version 9.3 was used for all the statistical analyses. Level of statistical significance was always set at α = 0.05.

### Ethics statement

The study was conducted according to the code of ethics from the Declaration of Helsinki. All participants provided a written informed consent and the study was approved by the Stockholm regional ethical committee (Dnr: 2010/1779-31/4).

## Results

The final sample (n = 165) had a mean age of 43.4 years (SD = 11.42 range 20–65), 67.3% were women and mean the MADRS score was 22.2 (SD = 6.89). Concurrent depressive and anxiety disorders were most common (73.6%), but part of the sample was exclusively suffering from either depressive (12.9%) or anxiety disorders (13.5%).

As shown in Table 2, the participants spent the largest proportion of their time being sedentary (546.4 minutes or 64.4% of total recorded time). On average 41.6 minutes were spent in MVPA and this accounted for 4.8% of their time. Just short of 20% of the sample fulfilled the recommended guidelines of doing MVPA for ≥30 minutes per day each week in bouts of ≥10 minutes. However, almost 34% fulfilled these requirements ≥5 days a week. A small part of the sample (12.1%, not shown in table) had no activity bouts, meaning that they did not do any MVPA that lasted ≥10 minutes during the observed week.

**Table 2:** Physical activity patterns as measured by accelerometer, by diagnosis and sex.

	**Counts per minute**	**Sedentary (minutes)**	**LIPA (minutes)**	**MVPA (minutes)**	**Total time in sedentary bouts (minutes)**	**Number of sedentary bouts**	**Time in activity bouts**	**Activity bouts, ≥30 min a day, ≥5 days a week %**	**Activity bouts, ≥30 min a day, 7 days a week %**
**Total sample (n = 165)**	630.69 (232.70)	546.41 (97.24)	260.35 (78.62)	41.55 (23.85)	230.62 (108.34)	6.41 (2.53)	18.16 (17.43)	33.94	19.39
**By diagnosis**									
***Depressive disorders (n = 21)***	546.64 (185.27)	579.82 (96.99)	235.97 (62.71)	37.75 (22.22)	251.40 (109.18)	7.03 (2.73)	15.07 (15.21)	28.57	14.29
***Concurrent disorders (n = 121)***	640.37 (233.34)	540.91 (95.29)	261.49 (77.90)	42.52 (24.96)	226.40 (104.11)	6.26 (2.34)	18.92 (17.70)	35.00	20.00
***Anxiety disorders (n = 22)***	648.59 (269.45)	552.12 (106.32)	269.25 (91.06)	40.95 (20.13)	240.02 (131.59)	6.76 (3.29)	18.33 (18.56)	36.36	22.73
**By sex**									
***Men (n = 54)***	619.4 (547.7)	566.0 (101.90)	**229.4 (72.74)**	45.24 (27.31)	**262.0 (105.50)**	**7.13 (2.39)**	20.57 (20.10)	40.74	22.22
***Women (n = 111)***	636.2 (595.2)	536.9 (93.87)	**275.4 (77.24)**	39.75 (21.89)	**215.4 (106.80)**	**6.06 (2.53)**	16.99 (15.94)	30.63	18.02

Numbers in **bold** represent statistically significant results (p<0.05) from two-tailed t-tests (sex) or ANOVA’s (diagnosis).

There were no statistically significant differences between diagnostic groups though there was a tendency for those categorized as purely depressed according to the MINI to be more sedentary and less active. This group spent the lowest number of minutes in both LIPA and MVPA but the highest number of minutes in sedentary behavior. They also spent less time in activity bouts than the other two groups, had a higher number of sedentary bouts and also spent more time on average in each sedentary bout. Those with concurrent disorders had less sedentary minutes than the other two groups and had more minutes in MVPA. They also had fewer sedentary bouts and they were on average shorter. Those categorized as having only anxiety disorders had the highest number of counts per minute and the highest number of minutes spent in LIPA,

The physical activity patterns of men and women were found to be statistically different in several instances; men spent more time in sedentary bouts and had one more sedentary bout on average than women. Women spent 46 more minutes in LIPA and although not statistically significant, men spent more time in MVPA and sedentary behavior.

Multiple linear regression models were used to explore the relationship between symptom severity (MADRS) and each of the continuous accelerometer variables (Table 3). Each one point increase in MADRS was associated with a 2.4 minute reduction in LIPA, independent of MVPA. A positive, though very weak, relationship between MADRS and number of sedentary bouts was found, meaning that a higher number of symptoms were associated with a higher number of bouts.

**Table 3:** The association between MADRS (independent) and each physical activity variable (dependent) as depicted by multiple linear regression models.

	**Model 1: MADRS, sex and age**		**Model 2: MADRS, sex, age, BMI and health status**		**Model 3: MADRS, sex, age, BMI, health status and MVPA**	
	**β**	**p**	**β**	**p**	**β**	**p**
**Counts per minute**	−1.58	0.555	−0.72	0.785	N/A*	N/A
**Sedentary (minutes)**	0.66	0.552	0.57	0.610	1.07	0.296
**LIPA (minutes)**	**−2.19**	**0.011**	**−1.98**	**0.021**	**−2.21**	**0.008**
**MVPA (minutes)**	0.23	0.403	0.29	0.295	N/A	N/A
**Time in sedentary bouts**	1.89	0.119	1.76	0.152	2.20	0.061
**Number of sedentary bouts**	0.05	0.085	0.04	0.124	**0.06**	**0.041**
**Time in activity bouts**	0.32	0.106	0.36	0.069	N/A*	N/A

*Counts per minute and time in activity bouts were not adjusted for MVPA as they were too correlated r>0.7. Numbers in **bold** represent statistically significant results (p<0.05).

## Discussion

In this study we found that persons with mild to moderate symptoms of depressive and/or anxiety disorders were sedentary the majority of their waking time (546 minutes or 9.1 hours) per day. This is considerably higher than estimates (459 minutes) from a previous population-based Swedish study which had similar protocols regarding the criteria for wear time and cut-off points for activity categories[26]. A difference of 87 minutes is quite substantial considering the detrimental effects of sedentary behavior on people’s general health (this difference is statistically significant, tested using an independent sample t-test p<0.05. Results from previous paper: 459 minutes, SD = 86, N = 1114, our results 546 minutes, SD = 97, N = 165)[14,15]. Our sample spent 260 minutes on average in LIPA, which is noticeably lower than the 340 minutes reported from the sample from the general Swedish population (the differences between the two values could not be tested as the standard deviation was not reported in the paper)[26]. Also relevant is that this previous study used uniaxial accelerometers and is therefore more likely to underestimate activity, which makes it probable that the differences are even larger than reported here.

There is evidence that exercise can reduce the symptoms of both anxiety[3,27,28] and depressive disorders[4,29]. Knowledge is currently limited regarding the optimal intensity for treating anxiety disorders but aerobic exercise seems to have a positive effect[27]. Regarding depression, a recent systematic review suggests that moderate intensity exercise (60–80% of maximum heart rate) is ideal[30] but this conclusion is based on the most common intensity prescribed in the interventions included in the review. As the whole sample spent very small proportions of their overall time in MVPA there is an opportunity to increase this activity and thereby treat their disorders.

The finding that there is an association between symptom severity (MADRS) and LIPA and sedentary bouts respectively, further suggest that interventions that target LIPA and sedentary behavior might be helpful. This is of importance to clinicians as it suggests that it might even be helpful to perform lighter forms of physical activity, such as taking walks, or changing sedentary behavior to shorter and fewer bouts, i.e. breaking up sedentary time. Some people are reluctant to start exercising at high intensity levels and it might therefore be of interest to encourage these people to begin by making small changes to their sedentary behavior by increasing LIPA and decreasing the number of sedentary bouts lasting ≥20 minutes.

Another interesting finding is the lack of difference in physical activity between disorders. Although one would expect that people suffering from anxiety would be more active and restless leading to them having a fewer sedentary bouts and spending more time in LIPA, the data does not support this assumption. There was however a tendency for those suffering only from depressive disorders to be less active than those who had comorbid disorders or only anxiety disorders. On the other hand, even though depressive and anxiety disorders are often conceptualized as if they were entirely separate disorders it is important to note that both disorders have a similar genetic profile and prevalence[31,32].This was evident in our study as the majority of the participants fulfilled the criteria for having both a depressive and an anxiety disorder.

The strength of this study lies in the fairly large sample of people with depressive and/or anxiety disorders and the measurement tools used to measure the variables of interest. Accelerometry is an objective way to measure physical activity and using a triaxial accelerometer allowed us to more accurately measure physical activity than is possible with a uniaxial model. Our cut-off points for activity categories and definitions of bouts were based on widely used protocols[22,24] which enhances comparability to previous studies. Additionally, the measurement tools for psychological symptoms, the PHQ-9[18,19], the MINI interview guide and the MADRS have been tested in previous studies and found to be reliable and valid[20,21].

A possible limitation is that only 53.6% of the sample actually wore their accelerometers for at least four days (including one weekend day). It is possible that we failed to detect some differences (for example between diagnoses) due to the small sample size. There were no differences in symptom severity (MADRS), or sex between those who wore their accelerometers and those that did not, however, both the age and BMI were significantly lower for non-wearers. This could have led to an underestimation of physical activity as younger and leaner people might be more active. However analyses of the physical activity in the year leading up to the inclusion in the study suggests otherwise as no statistically significant differences were found in this regard between wearers and non-wearers. The sample came from a randomized clinical trial, but the accelerometer data was collected prior to the commencement of treatment. It is therefore a convenience sample and this could give rise to volunteer bias. The cross-sectional nature of our data prevents us from drawing any conclusions regarding causality. It may very well be that the onset of either depressive or anxiety disorder reduces the individual’s tendency to be active which in turn might exasperate the symptoms. This vicious circle might be broken by exercising as previous studies have suggested[3,4,27–29] but the results of the current study suggest that future studies are needed to determine whether LIPA and number of sedentary bouts have a true biological effect or are merely a symptom of the disorder.

## Conclusions

The results of this study are highly relevant to public health and health care practitioners. The participants in this study were not so severely affected by their disorder to require inpatient care, but did need outpatient treatment. Physical exercise has been suggested as treatment for both depressive and anxiety disorders[3,4,27–29]. It is therefore important to investigate the patterns of physical activity of the persons affected so that it is possible to discern how they can be helped to increase their physical activity. The results of this study demonstrate that there is a large potential for treatment by changes in physical activity in people with mild to moderate depressive and anxiety disorders. The participants spent little time in MVPA in bouts of 10 minutes or more, and were in fact very sedentary, even more so than the general population. We also found that with increased symptom severity, LIPA decreased and sedentary bouts increased. Taken together these results imply that people might start increasing their LIPA and breaking up their sedentary time to alleviate symptoms of depressive and anxiety disorders, though this needs to be confirmed by studies with longitudinal designs. The results are especially encouraging to those patients who feel unequal to start strenuous exercise; instead they might start with these smaller changes and work their way up to more intense activities. To our knowledge, the current study is one of the first to use accelerometers to investigate daily physical activity patterns by assessing multiple aspects of physical activity in a non-clinical population.
